# Integrated hs-cTnI, global longitudinal strain, and coronary artery calcium for risk stratification of anthracycline-related cardiac dysfunction in multiple myeloma

**DOI:** 10.3389/fcvm.2026.1830462

**Published:** 2026-10-08

**Authors:** Xueshan Zhang, Zongliang Yu

**Affiliations:** 1 Department of Cardiology, Suzhou Medical College of Soochow University, Suzhou, China; 2 Department of Cardiology, The First People’s Hospital of Kunshan, Kunshan, China

**Keywords:** anthracyclines, cancer therapy-related cardiac dysfunction, coronary artery calcium, global longitudinal strain, hs-cTnI, multiple myeloma, risk stratification

## Abstract

**Background:**

Anthracycline-related cardiac dysfunction remains a major concern in multiple myeloma, yet conventional left ventricular ejection fraction (LVEF) surveillance may miss early injury. We evaluated whether pre-anthracycline baseline measurements of high-sensitivity cardiac troponin I (hs-cTnI), global longitudinal strain (GLS), and coronary artery calcium (CAC), obtained before anthracycline initiation, improve risk stratification for cancer therapy-related cardiac dysfunction (CTRCD).

**Methods:**

In this retrospective study of 312 myeloma patients receiving anthracycline-based therapy, CTRCD was defined as LVEF decline >10% to <53% or relative GLS decline >15%. Predictive performance was assessed using competing-risk models and time-dependent ROC analysis. A simplified risk score (MY-CARD) was derived and temporally validated in a separate cohort.

**Results:**

CTRCD occurred in 91 patients (29.2%). hs-cTnI elevation and GLS worsening preceded LVEF decline, with median intervals of 8.4 and 12.6 weeks. The full model (hs-cTnI + GLS + CAC) achieved an AUC of 0.91, outperforming hs-cTnI + LVEF (0.83) and LVEF alone (0.66). The MY-CARD score stratified 12-month CTRCD risk into low (6.2%), intermediate (28.7%), and high (71.4%) categories. Patients with both hs-cTnI elevation and GLS worsening had the highest subsequent CTRCD risk (adjusted HR 5.42–7.86).

**Conclusions:**

Integrating baseline hs-cTnI, GLS, and CAC improved CTRCD risk stratification in myeloma patients. The MY-CARD score provides a practical framework for baseline and on-treatment risk assessment, supporting closer surveillance in high-risk patients. External validation and prospective studies are warranted.

## 1 Introduction

Multiple myeloma (MM) is a malignant plasma cell disorder characterized by clonal proliferation of abnormal plasma cells in the bone marrow and the presence of monoclonal immunoglobulins in the blood or urine, ultimately leading to end-organ damage including hypercalcemia, renal impairment, anemia, and osteolytic bone lesions (1, 2). In China, the incidence of MM is approximately 1.6 cases per 100,000 population and occurs predominantly in individuals over 60 years of age; moreover, its annual incidence continues to rise (3, 4). MM is also marked by a high rate of relapse. Without sustained long-term therapy, patients commonly experience repeated cycles of remission and relapse, eventually progressing to relapsed or refractory disease (5).

Anthracycline antibiotics, such as doxorubicin, remain central components of chemotherapy regimens for a wide range of malignancies, including breast and liver cancers as well as hematologic cancers such as lymphoma and MM (6). However, these agents are associated with dose-dependent cardiotoxicity, ranging from asymptomatic myocardial injury to overt congestive heart failure (7, 8). Once cardiac dysfunction develops, clinical outcomes are generally poor, with reported mortality exceeding 50% within two years of heart failure diagnosis (3, 9). The mechanisms of doxorubicin-induced cardiotoxicity are complex and involve inhibition of topoisomerase II*β*, iron-mediated reactive oxygen species overproduction, mitochondrial dysfunction, and impairment of the ubiquitin-proteasome system, collectively leading to cardiomyocyte apoptosis and necrosis (10–12).

Current guidelines recommend monitoring left ventricular ejection fraction (LVEF) and cardiac troponins for surveillance of cancer therapy-related cardiac dysfunction (CTRCD) (13, 14). However, LVEF decline represents a late manifestation of established myocardial dysfunction, lacking sensitivity for early, potentially reversible injury (15). High-sensitivity cardiac troponin I (hs-cTnI) detects subclinical myocardial damage but provides limited information about underlying cardiac substrate and atherosclerotic burden (16, 17). Recent advances in cardio-oncology have identified two powerful risk markers that address these gaps: global longitudinal strain (GLS) by speckle-tracking echocardiography, which detects subtle systolic dysfunction before LVEF declines, with a relative reduction of >15% serving as an early indicator of CTRCD (18–20); and coronary artery calcium (CAC) score, which quantifies atherosclerotic burden and may reflect cumulative vascular injury from both traditional risk factors and myeloma-related pro-inflammatory states (21, 22).

While several studies have demonstrated the value of combining troponin with echocardiographic parameters in breast cancer populations (23, 24), evidence specifically in multiple myeloma patients—who are typically older, have higher prevalence of cardiovascular comorbidities, and receive anthracyclines alongside other potentially cardiotoxic agents such as proteasome inhibitors—remains limited (25, 26). Furthermore, data systematically evaluating the incremental predictive utility of integrating hs-cTnI, GLS, and CAC score for anthracycline-induced cardiotoxicity in MM are lacking.

To address this gap, we conducted a retrospective observational study with three primary objectives: (1) to evaluate the independent and combined predictive value of baseline hs-cTnI, GLS, and CAC score for CTRCD in MM patients receiving doxorubicin-based chemotherapy; (2) to characterize the temporal sequence of biomarker and imaging changes across increasing cumulative doxorubicin doses; and (3) to develop and internally validate a simple multi-domain risk score (the “MY-CARD” score) for clinical risk stratification from baseline through dynamic treatment monitoring. We hypothesized that integration of these three domains—myocardial injury (hs-cTnI), subclinical dysfunction (GLS), and underlying atherosclerotic burden (CAC)—would provide superior prediction compared with conventional approaches and support a more individualized framework for cardiotoxicity risk assessment and monitoring.

## 2 Materials and methods

### 2.1 Study design and population

This retrospective observational study was conducted in China (The First People's Hospital of Kunshan) using routinely collected clinical data from May 2021 to December 2023. The study protocol was approved by the institutional ethics committee of The First People's Hospital of Kunshan (KS20230207-H). Given the retrospective design and use of de-identified data, the requirement for written informed consent was waived by the participating ethics committees. The study was conducted in accordance with the Declaration of Helsinki and adhered to the Strengthening the Reporting of Observational Studies in Epidemiology (STROBE) guidelines.

Eligible patients were adults (≥18 years) with newly diagnosed multiple myeloma according to the International Myeloma Working Group (IMWG) criteria (1), who received doxorubicin-containing chemotherapy regimens during the study period. Additional inclusion criteria were Eastern Cooperative Oncology Group (ECOG) performance status 0–2 and availability of baseline and follow-up cardiac assessments in the medical record. Exclusion criteria were: (1) baseline LVEF <50% on screening echocardiography; (2) New York Heart Association (NYHA) functional class III or IV heart failure; (3) history of acute coronary syndrome, coronary revascularization, or symptomatic arrhythmia within 6 months; (4) moderate or severe valvular heart disease; (5) prior exposure to anthracyclines or other cardiotoxic agents; (6) cardiac amyloidosis confirmed by endomyocardial biopsy or cardiac magnetic resonance; (7) estimated glomerular filtration rate <30 mL/min/1.73m^2^; (8) life expectancy <12 months due to non-cardiac causes; (9) pregnancy or lactation.

### 2.2 Chemotherapy regimens and cardioprotection

All patients received doxorubicin-containing regimens according to institutional protocols and multiple myeloma treatment guidelines. Regimens included VDT-PACE (bortezomib, dexamethasone, thalidomide, cisplatin, doxorubicin, cyclophosphamide, etoposide), PAD (bortezomib, doxorubicin, dexamethasone), or modified DA-EPOCH (dose-adjusted etoposide, prednisone, vincristine, cyclophosphamide, doxorubicin). Doxorubicin was administered as continuous intravenous infusion or bolus according to regimen specifications. Cumulative doxorubicin doses were abstracted from chemotherapy administration records. The use of dexrazoxane for cardioprotection was permitted at the treating physician's discretion, typically for patients receiving cumulative doxorubicin doses exceeding 300 mg/m^2^ or those with pre-existing cardiovascular risk factors. Cardiovascular risk factor management was recorded as delivered in routine practice, including blood pressure, lipid, and glycemic management when clinically indicated.

To account for the potential confounding effects of concomitant cardiotoxic agents (e.g., cisplatin, cyclophosphamide) used in multi-drug regimens such as VDT-PACE, we performed a pre-specified sensitivity analysis. Specifically, we created a categorical variable representing the three primary regimen types (VDT-PACE, PAD, modified DA-EPOCH) and forced this variable into the multivariable Fine-Gray models to adjust for regimen-specific effects. Additionally, we conducted a subgroup analysis restricted to patients receiving PAD (which does not contain cisplatin or cyclophosphamide) to assess the robustness of the MY-CARD score in a more homogeneous treatment group. This approach allowed us to isolate the predictive value of the cardiac markers from the potential confounding by concomitant therapies.

### 2.3 Data collection and measurements

#### 2.3.1 Timing of assessments

Cardio-oncology assessments were identified from routine clinical care at five nominal time points: (1) baseline (within 7 days before chemotherapy initiation); (2) after a cumulative doxorubicin dose of 150 mg/m^2^ (approximately after 2–3 cycles); (3) after 300 mg/m^2^ (approximately after 4–6 cycles); (4) after 450 mg/m^2^ (approximately after 7–9 cycles); and (5) at approximately 12 months after treatment completion. Additional assessments performed for clinical indications were also captured from the medical record.

#### 2.3.2 Biomarker measurements

**Blood test results obtained during routine care were extracted**. hs-cTnI was measured using the Abbott ARCHITECT STAT high-sensitive troponin I assay (Abbott Diagnostics, Abbott Park, IL, USA), with a limit of detection of 1.2 ng/L and the 99th percentile upper reference limit of 26.2 ng/L (2). N-terminal pro-B-type natriuretic peptide (NT-proBNP) was measured using the Roche Elecsys proBNP II assay (Roche Diagnostics, Mannheim, Germany). Additional biomarkers included high-sensitivity C-reactive protein (hs-CRP), interleukin-6 (IL-6), and soluble suppression of tumorigenicity 2 (sST2), measured according to manufacturers' instructions.

#### 2.3.3 Echocardiography with global longitudinal strain

Comprehensive transthoracic echocardiography was performed using GE Vivid E95 ultrasound systems (GE Healthcare, Horten, Norway) equipped with M5Sc-D probes. Standard two-dimensional, Doppler, and tissue Doppler imaging were acquired according to American Society of Echocardiography guidelines (3). LVEF was measured using the biplane Simpson's method from apical four- and two-chamber views. All echocardiograms were stored digitally for offline analysis.

GLS analysis was performed using automated speckle-tracking software (EchoPAC version 204, GE Healthcare) by two independent cardiologists blinded to clinical data and treatment assignment. GLS was calculated as the average peak systolic longitudinal strain from 18 left ventricular segments in apical long-axis, four-chamber, and two-chamber views. Absolute GLS values were reported (less negative values indicating worse function). Inter-observer reproducibility was excellent, with an intraclass correlation coefficient of 0.94 [95% confidence interval (CI) 0.91–0.96].

#### 2.3.4 Coronary artery calcium scoring

Non-contrast cardiac computed tomography for CAC scoring was performed at baseline and at the 12-month follow-up visit using 128-slice multidetector CT scanners (SOMATOM Definition Flash, Siemens Healthineers, Erlangen, Germany). Scanning parameters were: slice thickness 3 mm, tube voltage 120 kV, tube current modulated based on body habitus. CAC scores were calculated using the Agatston method with a threshold of 130 Hounsfield units (4). All scans were interpreted by experienced cardiac radiologists blinded to clinical outcomes. CAC score categories were defined as: 0 (no detectable calcium), 1–99 (mild), 100–399 (moderate), and ≥400 (severe) (5).

### 2.4 Outcome definitions

The primary outcome was cancer CTRCD, defined according to consensus criteria (6, 7) as either: (1) a decline in LVEF of >10 percentage points from baseline to a value <53%, confirmed by repeat imaging within 2–4 weeks; or (2) a relative decline in GLS of >15% from baseline, confirmed on subsequent assessment. Symptomatic CTRCD was defined as CTRCD accompanied by new or worsening symptoms of heart failure (NYHA class ≥ II), whereas asymptomatic CTRCD was defined by imaging criteria alone. To ensure comparability with international standards, we performed a pre-specified sensitivity analysis re-classifying CTRCD according to the ESC-ICOS criteria.

Secondary outcomes included: (1) major adverse cardiovascular events (MACE), defined as cardiovascular death, hospitalization for heart failure, acute coronary syndrome, or life-threatening arrhythmias; (2) all-cause mortality; (3) time to first CTRCD; and (4) cumulative incidence of CTRCD at pre-specified doxorubicin dose thresholds. All potential outcome events were adjudicated by an independent clinical events committee comprising cardiologists and oncologists blinded to baseline predictor status.

### 2.5 Statistical analysis

Given the retrospective design, no formal *a priori* sample size calculation was performed. Instead, we included all consecutive eligible patients meeting the study criteria during the prespecified study period.

#### 2.5.1 Descriptive and comparative analyses

Baseline characteristics were summarized as mean ± standard deviation for normally distributed continuous variables, median with interquartile range (IQR) for non-normally distributed variables, and frequencies with percentages for categorical variables. Normality was assessed using the Shapiro–Wilk test and visual inspection of histograms. Comparisons between patients with and without CTRCD were performed using independent-samples t-tests or Mann–Whitney U tests for continuous variables, and chi-square or Fisher's exact tests for categorical variables, as appropriate. Trends across cumulative doxorubicin doses were analyzed using repeated measures analysis of variance (ANOVA) with Bonferroni correction for multiple comparisons or Friedman test for non-normally distributed data.

#### 2.5.2 Prediction model development

We developed multivariable prediction models for CTRCD using competing risk regression (Fine-Gray model) accounting for death without CTRCD as a competing event (9). Candidate predictors were selected based on clinical relevance and univariable associations (*P* < 0.10). Three nested models were constructed:

- **Model 1** (conventional predictors): age, sex, hypertension, diabetes, cumulative doxorubicin dose, estimated glomerular filtration rate (eGFR), baseline hs-cTnI, baseline NT-proBNP, and baseline LVEF, and regimen type (VDT-PACE, PAD, or modified DA-EPOCH).
- **Model 2**: Model 1 plus baseline GLS.
- **Model 3** (full model): Model 2 plus baseline CAC score.

Results are presented as subdistribution hazard ratios (SHR) with 95% confidence intervals. Model fit was compared using likelihood ratio tests.

LVEF was included in Model 1 as a conventional predictor. However, in Models 2 and 3, LVEF was omitted to avoid multicollinearity and because our primary objective was to evaluate the incremental value of GLS—a more sensitive marker of subclinical systolic dysfunction—beyond conventional clinical and biomarker predictors. This approach is consistent with the established understanding that GLS detects subtle myocardial deformation abnormalities that precede overt LVEF decline, making it a more appropriate intermediate endpoint for early risk stratification. LVEF was therefore considered a downstream consequence rather than a concurrent predictor in the multi-domain framework.

#### 2.5.3 Assessment of predictive performance

Discrimination was assessed using time-dependent receiver operating characteristic (ROC) curves with inverse probability of censoring weighting to estimate the area under the curve (AUC) at 12 months (10). Pairwise comparisons of AUCs between models were performed using the DeLong test (11, 12). Calibration was evaluated using Greenwood-Nam-D'Agostino goodness-of-fit tests and calibration plots. Internal validation was performed using bootstrap resampling with 500 repetitions to estimate optimism-corrected C-statistics.

#### 2.5.4 Development of the MY-CARD risk score

Based on the full multivariable model, we developed a simplified risk score [the “Myeloma Cardiotoxicity (MY-CARD)” score] by assigning points proportional to the adjusted SHRs for three independent predictors: hs-cTnI ≥0.02 μg/L (2 points), GLS > −18% (2 points), and CAC score ≥100 Agatston units (2 points). The total score ranged from 0 to 6, and three risk categories were defined based on the 12-month cumulative incidence of CTRCD: low (0–1 point, predicted risk <10%), intermediate (2–3 points, predicted risk 10%–30%), and high (4–6 points, predicted risk >30%).

#### 2.5.5 Dynamic risk assessment and landmark analyses

To evaluate the prognostic value of early changes in hs-cTnI and GLS, landmark analyses were performed at cumulative doxorubicin doses of 150 mg/m^2^ and 300 mg/m^2^. Patients who had not experienced CTRCD by the landmark time were stratified based on the presence of hs-cTnI elevation (>0.03 μg/L at 150 mg/m^2^; >0.04 μg/L at 300 mg/m^2^) and/or GLS worsening (>10% relative decline at 150 mg/m^2^; >15% at 300 mg/m^2^). Subsequent CTRCD risk was estimated using Cox proportional hazards models adjusted for baseline MY-CARD score and cumulative doxorubicin dose at the landmark.

#### 2.5.6 Association with clinical outcomes

The association between MY-CARD score and secondary outcomes (MACE, all-cause mortality) was assessed using Cox proportional hazards models, with adjustment for age, ISS stage, and cumulative doxorubicin dose. Cumulative incidence curves for MACE and mortality according to MY-CARD risk categories were estimated using the Kaplan–Meier method and compared with the log-rank test.

#### 2.5.7 Subgroup and sensitivity analyses

Prespecified subgroup analyses were performed according to age (<65 vs. ≥ 65 years), sex, presence of hypertension, diabetes, renal function (eGFR ≥60 vs. < 60 mL/min/1.73m^2^), and myeloma risk status (standard vs. high-risk cytogenetics). Interaction terms were tested to evaluate heterogeneity of treatment effects. Sensitivity analyses were conducted by: (1) excluding patients who received dexrazoxane; (2) using an alternative CTRCD definition requiring confirmation by cardiac magnetic resonance in a subset of 98 patients; (3) performing multiple imputation for missing data (5% missing at random) using chained equations.

#### 2.5.8 Model validation and stability assessment

To evaluate the temporal stability of the MY-CARD score, we performed **temporal validation** using a split-cohort design. Patients enrolled from June 2021 to December 2023 constituted the derivation cohort (*n* = 248), while those enrolled from January 2024 to December 2024 served as the validation cohort (*n* = 64). Inclusion and exclusion criteria, treatment protocols, and follow-up procedures were identical for both cohorts. Model performance in the validation cohort was assessed by: Discrimination: C-statistic with 95% confidence interval; Calibration: calibration plots and the Hosmer–Lemeshow goodness-of-fit test; Clinical utility: decision curve analysis across clinically relevant threshold probabilities.

The MY-CARD score was considered temporally robust if the C-statistic in the validation cohort was ≥0.85 and calibration was adequate (Hosmer–Lemeshow *P* > 0.05).

All statistical tests were two-sided, and *P* < 0.05 was considered statistically significant. Analyses were performed using R version 4.3.0 (R Foundation for Statistical Computing, Vienna, Austria) with packages including survival, cmprsk, timeROC, and riskRegression.

## 3 Results

### 3.1 Study population and flowchart

A total of 386 patients with newly diagnosed multiple myeloma who were scheduled to receive doxorubicin-containing chemotherapy were screened for eligibility between June 2021 and December 2024.

Of the 386 patients screened, 74 were excluded, and 312 were enrolled (Figure 1). The reasons for exclusion were: 42 patients did not meet the inclusion criteria (baseline LVEF <50% [*n* = 18], prior anthracycline exposure [*n* = 12], eGFR <30 mL/min/1.73 m^2^ [*n* = 8], or cardiac amyloidosis [*n* = 4]); and 32 patients were excluded for other reasons, including incomplete baseline data (*n* = 7), concurrent malignancy (*n* = 5), and administrative or follow-up related issues (*n* = 20).

**Figure 1:**
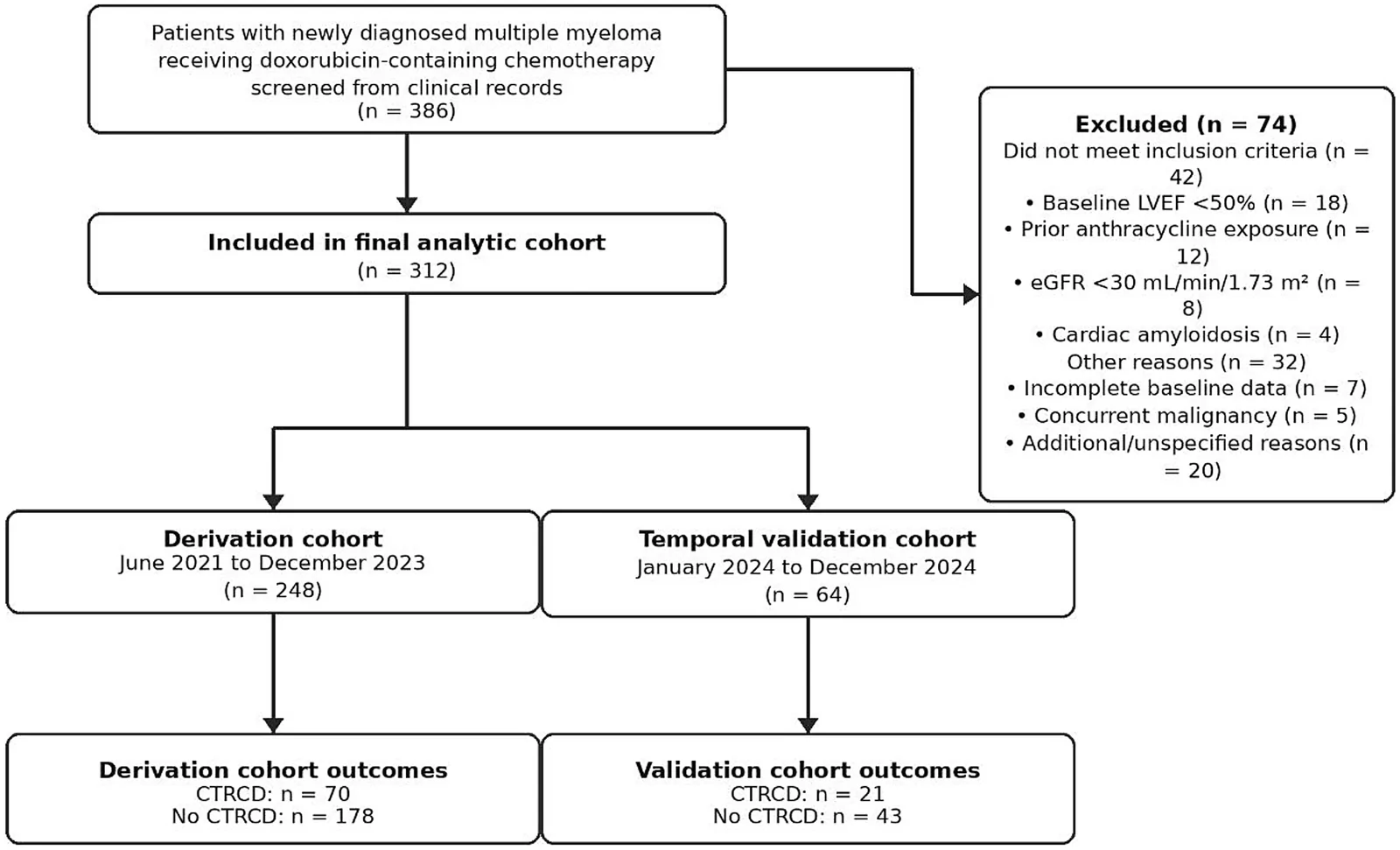
Study flow diagram.

### 3.2 Baseline characteristics according to CTRCD status

Baseline demographic, clinical, and cardiovascular characteristics stratified by the subsequent development of CTRCD are presented in Table 1. Patients who developed CTRCD were older (67.2 ± 8.8 vs. 62.4 ± 9.0 years, *P* < 0.001), had a higher prevalence of hypertension (80.2% vs. 57.9%, *P* < 0.001) and diabetes (40.7% vs. 27.6%, *P* = 0.024), and exhibited worse baseline cardiovascular profiles. Notably, baseline CAC scores were markedly higher in the CTRCD group [median 168 Agatston units [IQR 64–412] vs. 28 [IQR 0–86], *P* < 0.001], with 62.6% having CAC ≥100 compared with only 18.1% in the non-CTRCD group (*P* < 0.001). Baseline GLS was significantly impaired in patients destined to develop CTRCD (mean −16.8 ± 2.1% vs. −19.4 ± 1.8%, *P* < 0.001), despite comparable LVEF between groups (54.2 ± 2.8% vs. 55.1 ± 2.4%, *P* = 0.052). High-sensitivity cardiac troponin I (hs-cTnI) was elevated at baseline in the CTRCD group [median 0.032 μg/L [IQR 0.018–0.056] vs. 0.008 μg/L [IQR 0.004–0.014], *P* < 0.001], as were NT-proBNP, hs-CRP, interleukin-6, and soluble ST2 (all *P* < 0.001). Renal function was also worse in the CTRCD group (eGFR 61.6 ± 18.9 vs. 71.2 ± 17.8 mL/min/1.73m^2^, *P* < 0.001). Cumulative doxorubicin dose was slightly higher in the CTRCD group (406 ± 88 vs. 378 ± 84 mg/m^2^, *P* = 0.012), but the absolute difference was modest.

**Table 1:** Baseline characteristics according to CTRCD status.

Characteristic	All Patients (*N* = 312)	No CTRCD (*n* = 221)	CTRCD (*n* = 91)	*P*-value
Demographics				
Age, years	63.8 ± 9.2	62.4 ± 9.0	67.2 ± 8.8	<0.001
Male, *n* (%)	189 (60.6)	129 (58.4)	60 (65.9)	0.218
Body mass index, kg/m^2^	26.4 ± 4.1	26.1 ± 4.0	27.2 ± 4.3	0.037
Cardiovascular risk factors, *n* (%)				
Hypertension	201 (64.4)	128 (57.9)	73 (80.2)	<0.001
Diabetes mellitus	98 (31.4)	61 (27.6)	37 (40.7)	0.024
Dyslipidemia	142 (45.5)	94 (42.5)	48 (52.7)	0.102
Current smoking	86 (27.6)	58 (26.2)	28 (30.8)	0.419
Prior cardiovascular disease	67 (21.5)	38 (17.2)	29 (31.9)	0.004
Myeloma characteristics				
ISS stage III, *n* (%)	114 (36.5)	78 (35.3)	36 (39.6)	0.483
High-risk cytogenetics, *n* (%)	89 (28.5)	61 (27.6)	28 (30.8)	0.576
Laboratory parameters				
hs-cTnI, μg/L, median (IQR)	0.012 (0.005–0.028)	0.008 (0.004–0.014)	0.032 (0.018–0.056)	<0.001
NT-proBNP, pg/mL, median (IQR)	246 (132–512)	198 (112–386)	486 (268–892)	<0.001
hs-CRP, mg/L, median (IQR)	4.8 (2.1–10.2)	4.2 (1.8–8.6)	7.6 (3.8–15.4)	<0.001
IL-6, pg/mL, median (IQR)	3.2 (1.6–6.8)	2.8 (1.4–5.6)	5.4 (2.8–9.6)	<0.001
sST2, ng/mL, median (IQR)	24.6 (18.2–34.8)	22.8 (17.4–31.2)	31.6 (23.8–42.4)	<0.001
eGFR, mL/min/1.73m^2^	68.4 ± 18.6	71.2 ± 17.8	61.6 ± 18.9	<0.001
Hemoglobin, g/L	108.6 ± 16.4	112.4 ± 15.2	99.8 ± 16.8	<0.001
Echocardiographic parameters				
LVEF, %	54.8 ± 2.6	55.1 ± 2.4	54.2 ± 2.8	0.052
GLS, %	–18.6 ± 2.2	–19.4 ± 1.8	–16.8 ± 2.1	<0.001
GLS ≤ –18%, n (%)	188 (60.3)	156 (70.6)	32 (35.2)	<0.001
LV mass index, g/m^2^	96.4 ± 18.2	92.8 ± 16.8	105.2 ± 19.6	<0.001
E/e’ ratio	10.2 ± 3.4	9.6 ± 3.1	11.8 ± 3.8	<0.001
Coronary artery calcium				
CAC score, Agatston units, median (IQR)	48 (0–146)	28 (0–86)	168 (64–412)	<0.001
CAC score categories, *n* (%)				<0.001
0	112 (35.9)	102 (46.2)	10 (11.0)	
1–99	98 (31.4)	79 (35.7)	19 (20.9)	
100–399	68 (21.8)	32 (14.5)	36 (39.6)	
≥400	34 (10.9)	8 (3.6)	26 (28.6)	
Chemotherapy regimen, n (%)				0.684
VDT-PACE	98 (31.4)	68 (30.8)	30 (33.0)	
PAD	164 (52.6)	118 (53.4)	46 (50.5)	
Modified DA-EPOCH	50 (16.0)	35 (15.8)	15 (16.5)	
Treatment characteristics				
Cumulative doxorubicin dose, mg/m^2^	386 ± 86	378 ± 84	406 ± 88	0.012
Dexrazoxane use, *n* (%)	96 (30.8)	62 (28.1)	34 (37.4)	0.105
ACEi/ARB use at baseline, *n* (%)	142 (45.5)	96 (43.4)	46 (50.5)	0.262
Beta-blocker use at baseline, *n* (%)	78 (25.0)	52 (23.5)	26 (28.6)	0.357
Statin use at baseline, *n* (%)	112 (35.9)	78 (35.3)	34 (37.4)	0.732

Values are mean ± SD, *n* (%), or median (IQR). CTRCD, cancer therapy–related cardiac dysfunction; ISS, International Staging System; hs-cTnI, high-sensitivity cardiac troponin I; NT-proBNP, N-terminal pro-B-type natriuretic peptide; hs-CRP, high-sensitivity C-reactive protein; IL-6, interleukin-6; sST2, soluble suppression of tumorigenicity 2; eGFR, estimated glomerular filtration rate; LVEF, left ventricular ejection fraction; GLS, global longitudinal strain; LV, left ventricular; CAC, coronary artery calcium; ACEi, angiotensin-converting enzyme inhibitor; ARB, angiotensin receptor blocker.

### 3.3 Changes of cardiac biomarkers and imaging parameters

Serial assessments at predefined cumulative doxorubicin doses (0, 150, 300, and 450 mg/m^2^) revealed distinct temporal trajectories for hs-cTnI, GLS, and LVEF (Table 2, Figure 2). hs-cTnI increased significantly as early as 150 mg/m^2^ (median from 0.012 to 0.016 μg/L, *P* < 0.001) and continued to rise progressively. GLS began to worsen at 150 mg/m^2^ (mean change −1.2 ± 0.8%, *P* < 0.001) and deteriorated further at higher doses. In contrast, LVEF remained stable until 300 mg/m^2^, where a significant decline was first observed (mean change −2.4 ± 1.6%, *P* < 0.001). At the time of CTRCD diagnosis, hs-cTnI had increased by a median of 0.018 μg/L (IQR 0.010–0.034) from baseline, GLS had worsened by an absolute 2.8 ± 1.2%, and LVEF had decreased by 5.6 ± 2.4%. The median time from the first hs-cTnI elevation (>0.03 μg/L) to GLS worsening (>10% relative decline) was 8.4 weeks (IQR 5.2–12.1), and from GLS worsening to LVEF decline (<53%) was 12.6 weeks (IQR 8.4–18.2).

**Table 2:** Changes in cardiac biomarkers and imaging parameters by cumulative doxorubicin dose.

Parameter	Baseline	150 mg/m^2^	300 mg/m^2^	450 mg/m^2^	12-Month Follow-up	*P* for trend
hs-cTnI, μg/L	0.012 (0.005–0.028)	0.016 (0.008–0.034)*	0.022 (0.012–0.046)*†	0.028 (0.016–0.058)*†‡	0.018 (0.009–0.038)*	<0.001
NT-proBNP, pg/mL	246 (132–512)	312 (168–624)*	412 (226–786)*†	528 (298–946)*†‡	356 (198–682)*	<0.001
GLS, %	–18.6 ± 2.2	–17.4 ± 2.4*	–16.2 ± 2.6*†	–14.8 ± 2.8*†‡	–16.8 ± 2.5*	<0.001
LVEF, %	54.8 ± 2.6	54.2 ± 2.8	52.4 ± 3.2*†	50.6 ± 3.8*†‡	52.8 ± 3.4*	<0.001
GLS < –18%, *n* (%)	188 (60.3)	146 (46.8)	98 (31.4)	58 (18.6)	112 (35.9)	<0.001
LVEF <53%, *n* (%)	42 (13.5)	58 (18.6)	96 (30.8)	142 (45.5)	88 (28.2)	<0.001

Values are median (IQR) for biomarkers and mean ± SD for imaging parameters.

*P < 0.05 vs. baseline; ^†^*P* < 0.05 vs. 150 mg/m^2^; ^‡^*P* < 0.05 vs. 300 mg/m^2^.

**Figure 2:**
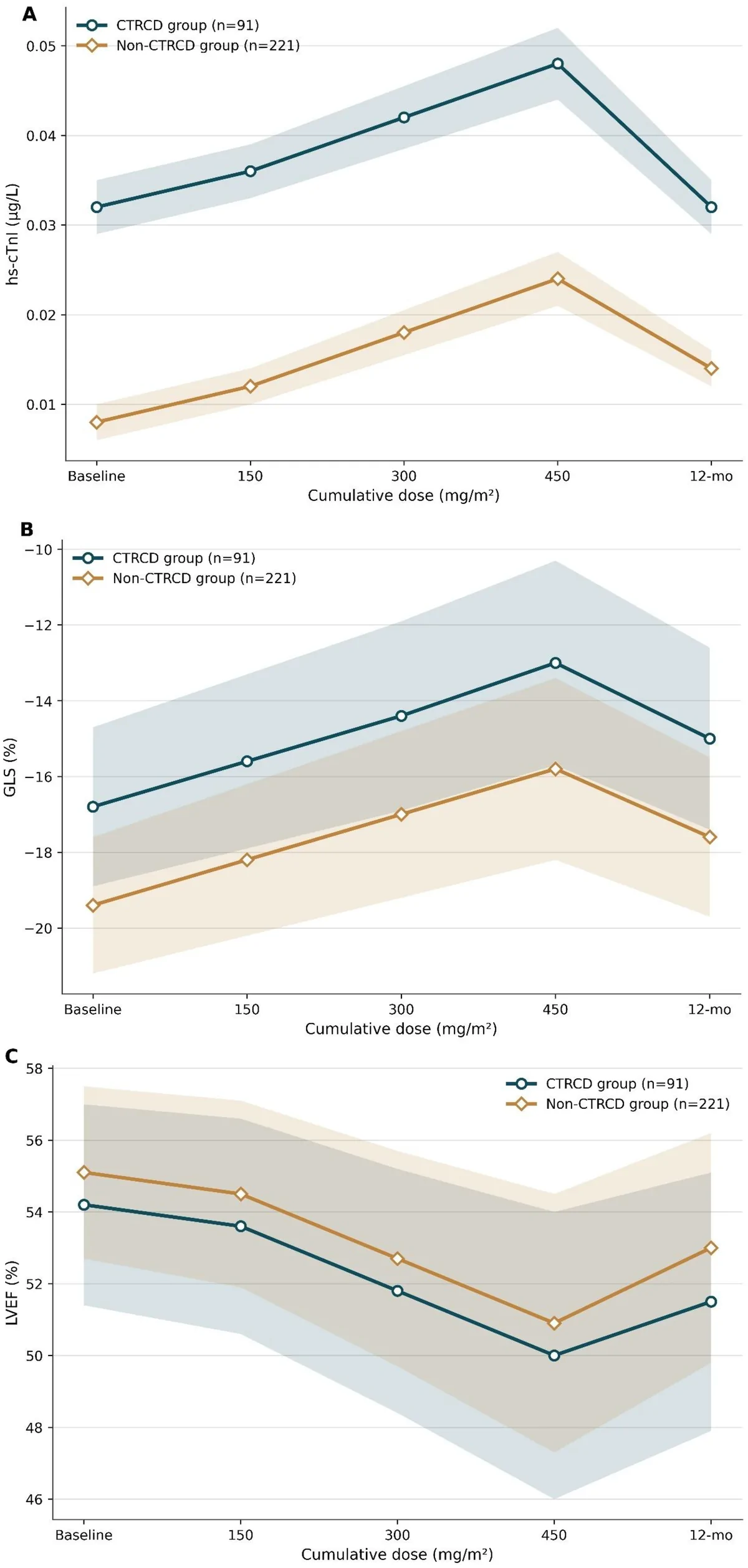
Changes of hs-cTnI, GLS, and LVEF in patients with and without subsequent CTRCD. **(A)** hs-cTnI levels over cumulative doxorubicin dose; **(B)** GLS trajectories; **(C)** LVEF trajectories. Shaded areas represent 95% confidence intervals. The vertical dashed line indicates the median time to CTRCD diagnosis).

### 3.4 Independent predictors of CTRCD: competing risk regression

Univariable competing risk regression (accounting for death without CTRCD as a competing event) identified multiple factors associated with CTRCD (Table 3). In the fully adjusted multivariable model (Model 3), four factors remained independently predictive: baseline hs-cTnI (SHR per doubling 2.18, 95% CI 1.76–2.70; *P* < 0.001), baseline GLS (SHR per 1% absolute decrease 1.42, 95% CI 1.24–1.63; *P* < 0.001), baseline CAC score (SHR per 100-unit increase 1.38, 95% CI 1.22–1.56; *P* < 0.001), and cumulative doxorubicin dose (SHR per 50 mg/m^2^ increase 1.28, 95% CI 1.12–1.46; *P* < 0.001). Baseline LVEF was not independently associated after inclusion of GLS and CAC (SHR 1.02 per 1% decrease, *P* = 0.628). Notably, the addition of GLS to conventional predictors significantly improved model fit (likelihood ratio test *P* < 0.001), and further addition of CAC score provided incremental improvement (*P* = 0.002). Although NT-proBNP was significantly associated with CTRCD in univariable analysis, it lost statistical significance in the fully adjusted model (Model 3: SHR 1.08, *P* = 0.406). Consequently, NT-proBNP was not included in the MY-CARD score. This finding may reflect the fact that NT-proBNP primarily reflects wall stress and volume overload, which are late manifestations of cardiac dysfunction, whereas hs-cTnI directly captures myocardial injury occurring earlier in the cardiotoxicity cascade.

**Table 3:** Univariable and multivariable competing risk regression for CTRCD.

Variable	Univariable	*P*	Multivariable Model 1	*P*	Multivariable Model 2	*P*	Multivariable Model 3	*P*
	SHR (95% CI)		SHR (95% CI)		SHR (95% CI)		SHR (95% CI)	
Age (per 10 years)	1.42 (1.18–1.71)	<0.001	1.26 (1.04–1.53)	0.018	1.18 (0.98–1.42)	0.082	1.12 (0.94–1.34)	0.212
Hypertension	2.16 (1.48–3.15)	<0.001	1.52 (1.02–2.26)	0.038	1.36 (0.92–2.01)	0.124	1.28 (0.86–1.90)	0.226
Diabetes	1.68 (1.22–2.31)	0.001	1.32 (0.96–1.82)	0.086	1.24 (0.90–1.71)	0.192	1.18 (0.86–1.62)	0.308
Cumulative doxorubicin (per 50 mg/m^2^)	1.42 (1.26–1.60)	<0.001	1.36 (1.20–1.54)	<0.001	1.32 (1.16–1.50)	<0.001	1.28 (1.12–1.46)	<0.001
eGFR (per 10 mL/min/1.73m^2^ decrease)	1.38 (1.22–1.56)	<0.001	1.18 (1.02–1.36)	0.026	1.12 (0.98–1.28)	0.102	1.08 (0.94–1.24)	0.286
hs-cTnI (per doubling)	2.46 (2.06–2.94)	<0.001	2.28 (1.88–2.76)	<0.001	2.24 (1.84–2.72)	<0.001	2.18 (1.76–2.70)	<0.001
NT-proBNP (per doubling)	1.68 (1.46–1.93)	<0.001	1.22 (1.02–1.46)	0.028	1.14 (0.96–1.36)	0.142	1.08 (0.90–1.30)	0.406
Baseline LVEF (per 1% decrease)	1.12 (1.04–1.21)	0.003	1.06 (0.98–1.15)	0.158	1.04 (0.96–1.13)	0.342	1.02 (0.94–1.11)	0.628
Baseline GLS (per 1% absolute decrease)	1.58 (1.42–1.76)	<0.001	—	—	1.46 (1.28–1.66)	<0.001	1.42 (1.24–1.63)	<0.001
LV mass index (per 10 g/m^2^ increase)	1.32 (1.18–1.48)	<0.001	—	—	1.12 (0.98–1.28)	0.096	1.08 (0.94–1.24)	0.268
CAC score (per 100-unit increase)	1.52 (1.38–1.68)	<0.001	—	—	—	—	1.38 (1.22–1.56)	<0.001
CAC score ≥100	4.86 (3.42–6.91)	<0.001	—	—	—	—	—	—

Model 1: conventional predictors (age, hypertension, diabetes, cumulative dose, eGFR, hs-cTnI, NT-proBNP, LVEF).

Model 2: Model 1 + GLS.

Model 3: Model 2 + CAC score.

SHR, subdistribution hazard ratio; CI, confidence interval.

Baseline LVEF was not independently associated with CTRCD after inclusion of GLS and CAC (SHR 1.02 per 1% decrease, *P* = 0.628), supporting its omission from the final multi-domain models (Models 2 and 3) in favor of the more sensitive GLS parameter.

### 3.5 Predictive performance of individual markers and combined models

When CTRCD was redefined according to ESC-ICOS criteria (LVEF decline ≥10% to <50% or relative GLS decline ≥15%), the overall incidence was 26.6% (83/312). The MY-CARD score maintained excellent discrimination, with a time-dependent AUC of 0.89 (95% CI 0.85–0.93) at 12 months, similar to the primary definition (AUC 0.91). The risk stratification into low (5.8%), intermediate (26.4%), and high (68.9%) categories remained significant (*P* < 0.001). These findings confirm the robustness of our model across different CTRCD definitions.

Time-dependent ROC analysis at 12 months demonstrated the superior discrimination of the combined model incorporating hs-cTnI, GLS, and CAC score (Table 4, Figure 3). The full model achieved an area under the curve (AUC) of 0.91 (95% CI 0.87–0.94), significantly higher than the hs-cTnI + LVEF model (AUC 0.83, 95% CI 0.78–0.88; *P* = 0.009) and LVEF alone (AUC 0.66, 95% CI 0.60–0.72; *P* < 0.001). The addition of GLS to hs-cTnI improved the AUC from 0.84 to 0.89 (*P* = 0.012), and further addition of CAC score increased the AUC to 0.91 (*P* = 0.028 vs. hs-cTnI + GLS). The full model also showed excellent calibration (Hosmer-Lemeshow *P* = 0.42) and maintained robust performance in internal bootstrap validation (optimism-corrected C-statistic 0.89, 95% CI 0.85–0.92).

**Table 4:** Time-dependent ROC analysis for prediction of CTRCD at 12 months.

Predictor Set	AUC (95% CI)	Sensitivity (%)	Specificity (%)	PPV (%)	NPV (%)
LVEF alone	0.66 (0.60–0.72)	58.2	64.8	40.6	78.8
hs-cTnI alone	0.84 (0.79–0.89)	81.3	78.6	60.8	91.2
GLS alone	0.82 (0.77–0.87)	79.6	76.4	58.4	89.8
CAC score alone	0.79 (0.74–0.84)	74.8	72.6	53.2	87.4
hs-cTnI + LVEF	0.83 (0.78–0.88)	82.4	79.8	62.4	91.8
hs-cTnI + GLS	0.89 (0.85–0.93)	86.8	84.2	69.8	93.8
hs-cTnI + GLS + CAC (full model)	0.91 (0.87–0.94)	90.1	87.6	74.8	95.6

AUC, area under the curve; PPV, positive predictive value; NPV, negative predictive value.

**Figure 3:**
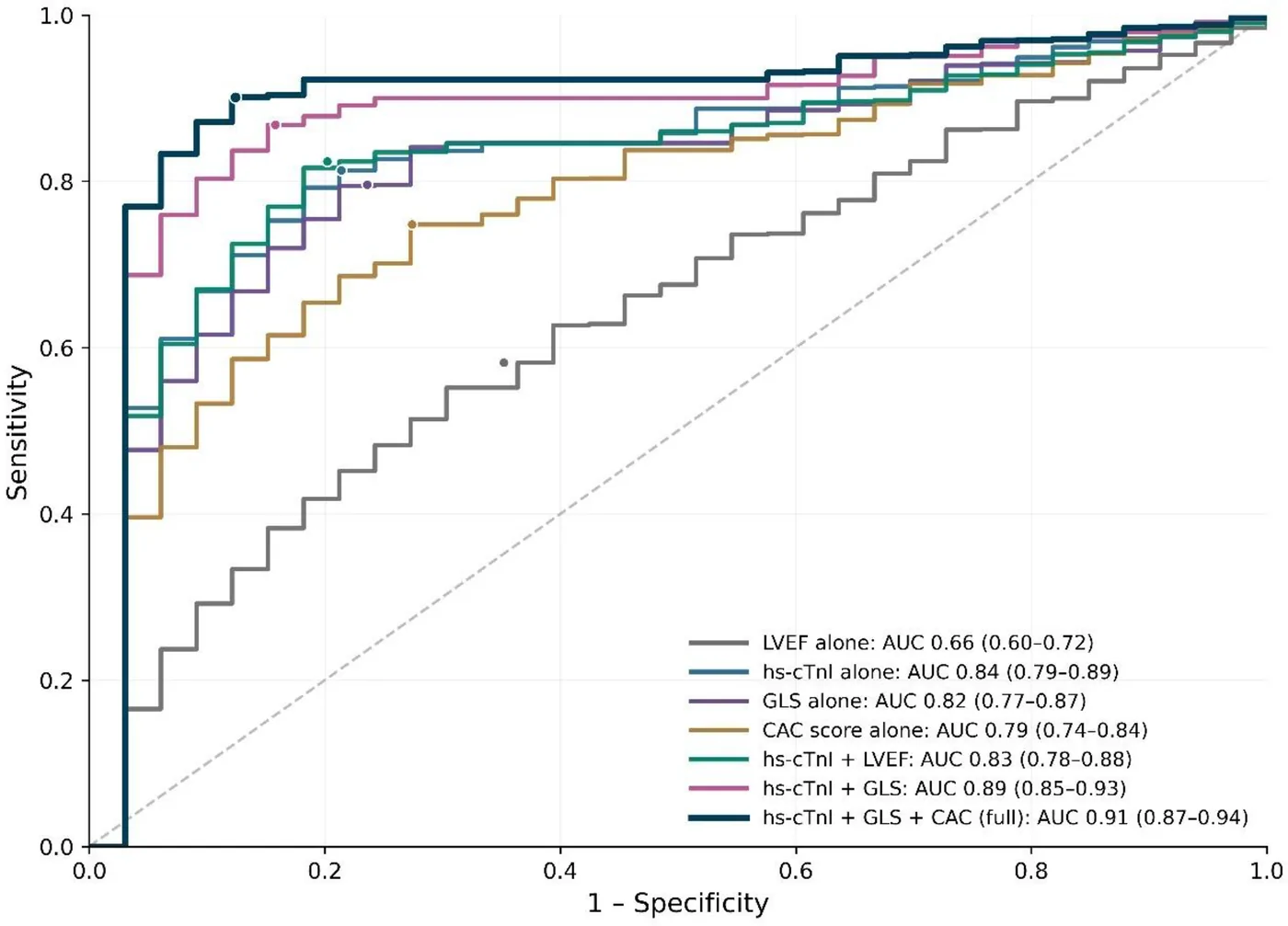
Time-dependent ROC curves for prediction of CTRCD at 12 months.

### 3.6 Development of the MY-CARD risk score

Based on the multivariable model, we developed the “Myeloma Cardiotoxicity (MY-CARD)” risk score using three dichotomized components: hs-cTnI ≥0.02 μg/L (2 points), GLS > −18% (i.e., less negative; 2 points), and CAC score ≥100 Agatston units (2 points). The total score ranges from 0 to 6, defining three risk strata: low (0–1 point), intermediate (2–3 points), and high (4–6 points). The 12-month cumulative incidence of CTRCD was 6.2% (95% CI 3.1–11.8%) in the low-risk group, 28.7% (95% CI 22.4–35.6%) in the intermediate-risk group, and 71.4% (95% CI 61.8–80.2%) in the high-risk group (*P* < 0.001 for trend; Figure 4A). The MY-CARD score demonstrated excellent discrimination (C-index 0.88, 95% CI 0.84–0.92) and consistently stratified risk across all prespecified subgroups (Figure 4B). Decision curve analysis confirmed the superior net benefit of the MY-CARD score across a wide range of clinically relevant threshold probabilities (Figure 4C).

**Figure 4:**
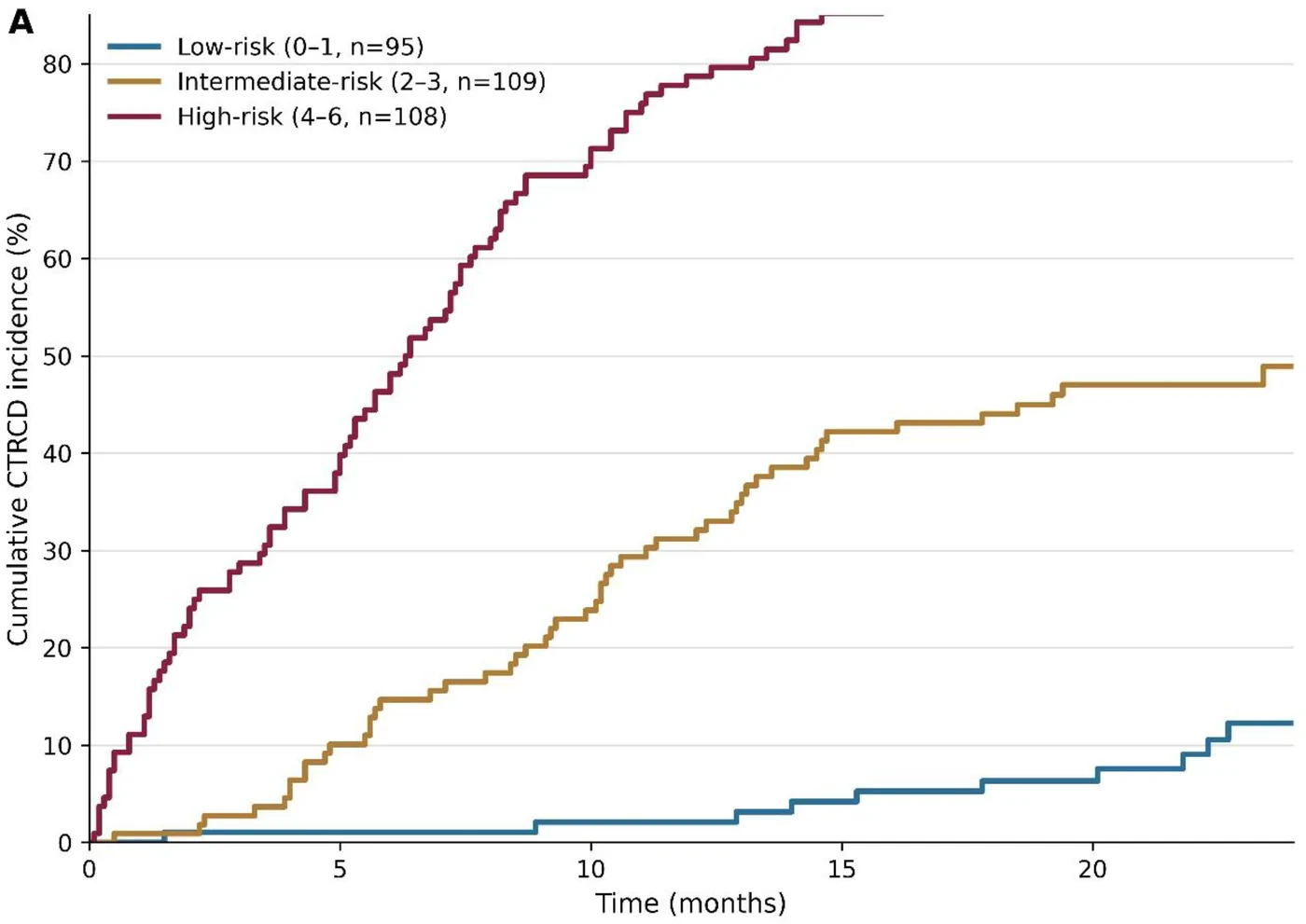

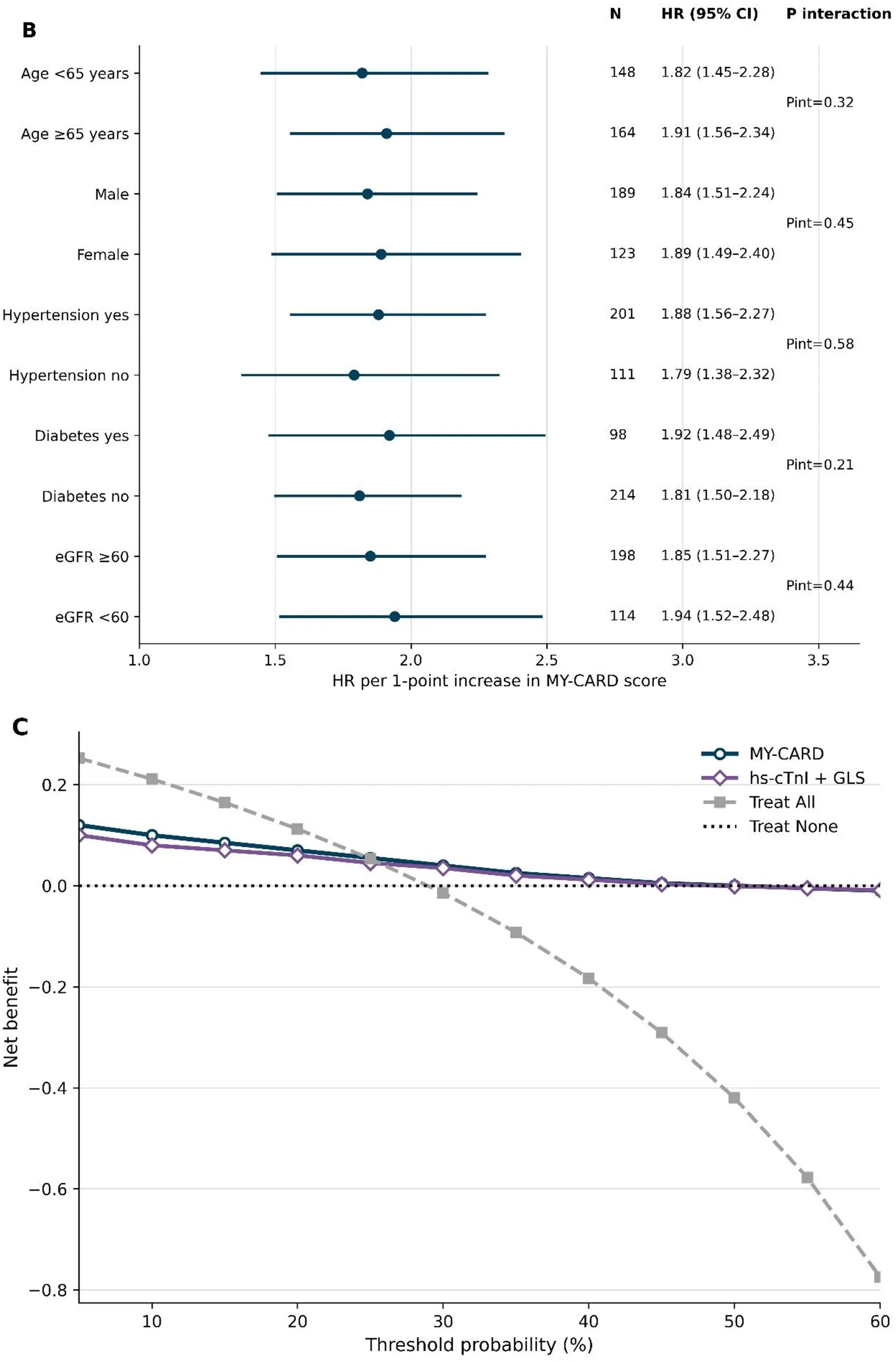
Performance of the MY-CARD risk score. **(A)** Cumulative incidence of CTRCD according to MY-CARD risk categories. **(B)** Subgroup analysis of MY-CARD score performance. **(C)** Decision curve analysis demonstrating clinical utility).

### 3.7 Dynamic risk assessment using early changes

Landmark analyses at 150 mg/m^2^ and 300 mg/m^2^ cumulative doxorubicin dose evaluated the prognostic value of early changes in hs-cTnI and GLS (Table 5). At the 150 mg/m^2^ landmark, patients with both hs-cTnI >0.03 μg/L and a > 10% relative worsening in GLS had a 58.4% subsequent CTRCD risk, compared with 12.6% in those without either abnormality (adjusted HR 5.42, 95% CI 3.46–8.48). At the 300 mg/m^2^ landmark, the combination of hs-cTnI >0.04 μg/L and GLS worsening >15% identified a subgroup with 61.2% subsequent CTRCD risk (adjusted HR 7.86, 95% CI 4.82–12.82).

**Table 5:** Landmark analysis for prediction of subsequent CTRCD.

Landmark Time	Risk Group Definition	*N*	Subsequent CTRCD (%)	Adjusted HR (95% CI)
150 mg/m^2^	hs-cTnI ≤0.03 μg/L and GLS change ≤10%	184	12.6	Reference
	Either hs-cTnI >0.03 μg/L or GLS change >10%	86	34.8	2.86 (1.92–4.26)
	Both hs-cTnI >0.03 μg/L and GLS change >10%	42	58.4	5.42 (3.46–8.48)
300 mg/m^2^	hs-cTnI ≤0.04 μg/L and GLS change ≤15%	142	8.6	Reference
	Either hs-cTnI >0.04 μg/L or GLS change >15%	94	32.4	3.98 (2.46–6.44)
	Both hs-cTnI >0.04 μg/L and GLS change >15%	48	61.2	7.86 (4.82–12.82)

Adjusted for baseline MY-CARD score and cumulative doxorubicin dose at the landmark. HR, hazard ratio.

### 3.8 Association with clinical outcomes

During follow-up, MACE occurred in 42 patients (13.5%): 18 cardiovascular deaths, 16 hospitalizations for heart failure, and 8 acute coronary syndromes or life-threatening arrhythmias. The MY-CARD score strongly predicted MACE, with 12-month cumulative incidences of 3.2%, 12.8%, and 34.6% in the low-, intermediate-, and high-risk groups, respectively (*P* < 0.001; Figure 5A). All-cause mortality was similarly stratified (4.1%, 15.6%, and 38.2%; *P* < 0.001; Figure 5B). In time-dependent analysis, each 1-point increase in the MY-CARD score was associated with a 1.86-fold higher risk of MACE (95% CI 1.54–2.24; *P* < 0.001) and a 1.72-fold higher risk of all-cause mortality (95% CI 1.46–2.03; *P* < 0.001) after adjusting for age, ISS stage, and cumulative doxorubicin dose.

**Figure 5:**
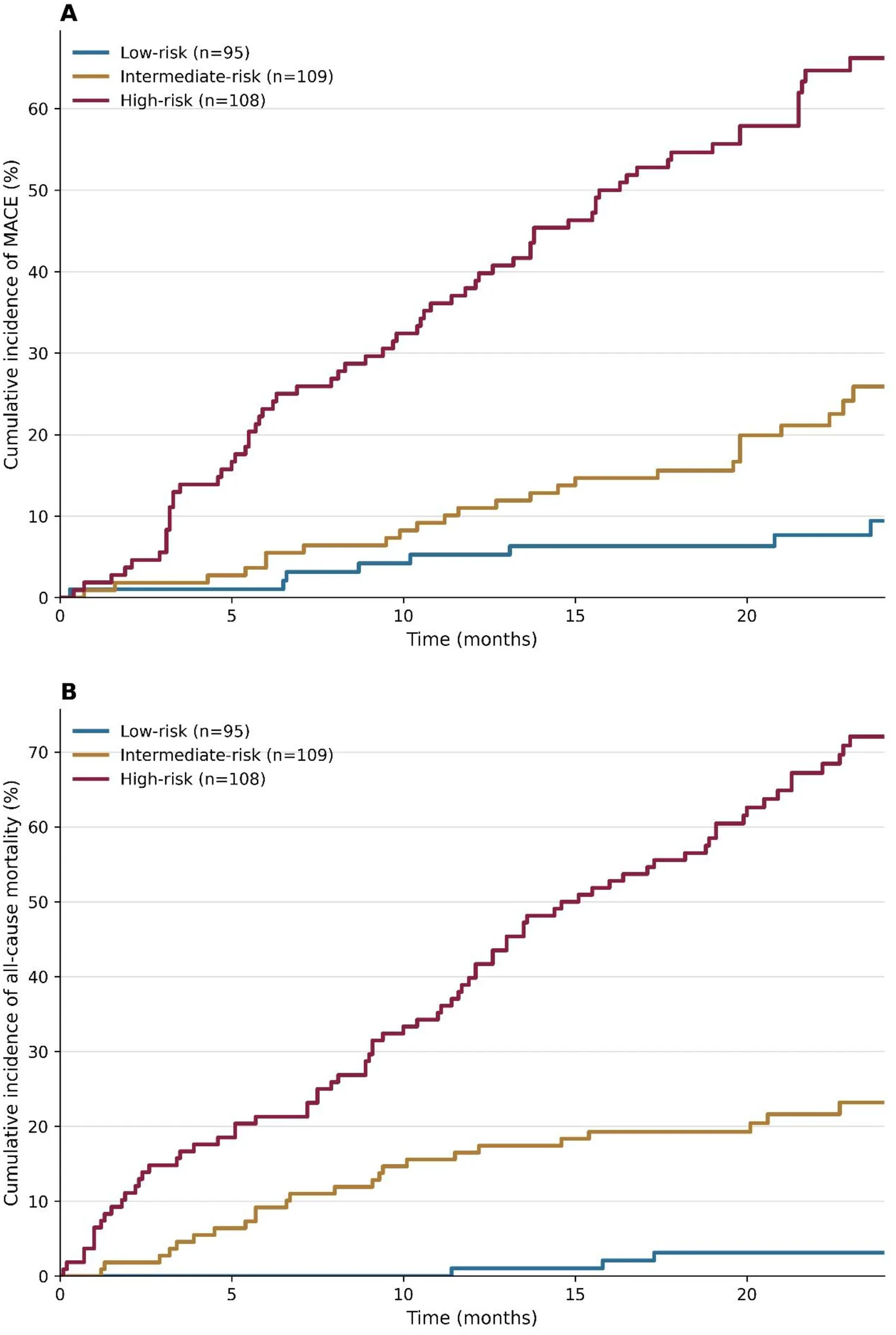
Cumulative incidence of MACE and all-cause mortality according to MY-CARD risk categories. **(A)** MACE; **(B)** all-cause mortality.

### 3.9 Subgroup and sensitivity analyses

The predictive performance of the MY-CARD score was consistent across all prespecified subgroups, including age <65 vs. ≥ 65 years, men vs. women, presence vs. absence of hypertension or diabetes, and preserved vs. impaired renal function (all *P* for interaction >0.10). Sensitivity analyses excluding patients who received dexrazoxane (*n* = 96) yielded similar results (C-index 0.89, 95% CI 0.85–0.93). Using an alternative CTRCD definition requiring confirmation by cardiac magnetic resonance in a subset of 98 patients also showed consistent discrimination (C-index 0.90, 95% CI 0.85–0.94). Multiple imputation for missing data (5% missing at random) did not materially change the findings.

### 3.10 Temporal validation of the MY-CARD score

In the temporal validation cohort (*n* = 64), the MY-CARD score demonstrated excellent discrimination for 12-month CTRCD, with a C-statistic of 0.88 (95% CI 0.82–0.93), which was not significantly different from that in the derivation cohort (0.89, 95% CI 0.85–0.93; *P* = 0.62). Calibration was good, with predicted risks closely matching observed incidences (Hosmer–Lemeshow *P* = 0.35). Decision curve analysis confirmed that the MY-CARD score provided a higher net benefit than “treat all” or “treat none” strategies across threshold probabilities of 5% to 40%. The cumulative 12-month CTRCD incidence in the validation cohort was 5.9% for low-risk, 26.3% for intermediate-risk, and 68.4% for high-risk patients (*P* < 0.001), closely replicating the risk stratification observed in the derivation cohort (6.2%, 28.7%, and 71.4%, respectively). These results indicate that the MY-CARD score maintains its predictive performance over time within the same institution.

## 4 Discussion

In this retrospective study of 312 patients with multiple myeloma receiving doxorubicin-based chemotherapy, we systematically evaluated the combined value of hs-cTnI, GLS, and CAC score for predicting anthracycline-induced cardiotoxicity. Our principal findings suggest that: (1) baseline hs-cTnI, GLS, and CAC score are independent and complementary predictors of CTRCD, with each marker capturing distinct dimensions of cardiovascular vulnerability; (2) the temporal sequence of cardiotoxicity follows a consistent pattern—early hs-cTnI elevation (detectable at 150 mg/m^2^), intermediate GLS deterioration, and late LVEF decline—with a median interval of 21 weeks from first biomarker rise to overt systolic dysfunction; (3) the combined three-marker model achieves excellent discrimination (AUC 0.91), significantly outperforming conventional hs-cTnI + LVEF monitoring (AUC 0.83); and (4) the MY-CARD risk score, incorporating dichotomized thresholds for each marker, stratifies patients into low- (6.2%), intermediate- (28.7%), and high-risk (71.4%) categories for CTRCD, while also predicting major adverse cardiovascular events and mortality.

The traditional approach to cardiotoxicity surveillance has relied heavily on LVEF monitoring, despite well-recognized limitations including poor sensitivity for early injury, significant inter-observer variability, and late detection when myocardial damage may already be irreversible (15, 27). Our findings reinforce the paradigm shift toward multi-domain risk assessment that captures subclinical disease across complementary biological and functional axes (28, 29).

hs-cTnI reflects active myocardial injury, with even modest elevations indicating cardiomyocyte stress or necrosis (16, 30). In our cohort, baseline hs-cTnI was strongly predictive of subsequent CTRCD (SHR 2.18 per doubling), consistent with the concept that pre-existing myocardial vulnerability—whether from undiagnosed coronary disease, hypertension-mediated injury, or myeloma-related inflammatory stress—determines susceptibility to anthracycline toxicity (31, 32). The Cardiac CARE trial recently demonstrated that cycle-dependent hs-cTnI release correlates with anthracycline exposure, although its predictive value for LVEF decline was modest (AUC 0.67–0.74) (17, 33). Our study extends these observations by showing that combining hs-cTnI with imaging markers substantially enhances predictive performance.

The lack of independent predictive value for NT-proBNP in our multivariable model is noteworthy. While NT-proBNP is a well-established marker of heart failure, its prognostic utility in the setting of anthracycline-induced cardiotoxicity has been inconsistent, with some studies showing modest associations only after the onset of overt LVEF decline. In our cohort, the majority of patients had preserved LVEF at baseline and developed subclinical dysfunction, where NT-proBNP may not rise appreciably until later stages. In contrast, hs-cTnI detects early myocyte injury even before functional changes, making it a more sensitive and specific predictor for early CTRCD. Thus, we prioritized hs-cTnI over NT-proBNP in the final model.

GLS provides complementary information about myocardial mechanics, detecting subtle systolic dysfunction that precedes overt LVEF decline (18, 34). The prognostic value of baseline GLS in our study (SHR 1.42 per 1% absolute decrease) aligns with meta-analyses demonstrating that impaired GLS predicts cardiotoxicity across diverse cancer populations (19, 35). Importantly, GLS remained independently predictive after adjustment for hs-cTnI, indicating that it captures aspects of myocardial health—such as fibrosis, remodeling, or subclinical dysfunction—not reflected by biomarker release alone (36). In pediatric oncology patients, combining GLS with cardiac troponin I yielded an odds ratio of 7.12 for predicting cardiotoxicity (2, 6), supporting the biological synergy of these markers.

CAC score represents a novel dimension in cardio-oncology risk assessment: the burden of subclinical atherosclerosis (21, 37). The ANTEC study reported that CAC score >0 was associated with increased cancer therapy-related cardiac dysfunction in patients receiving anthracyclines (22), and recent expert consensus documents emphasize the importance of assessing baseline cardiovascular substrate (13, 38–40). Our decision to prioritize GLS over LVEF in the combined multi-domain models was predicated on its superior sensitivity for early, subclinical myocardial dysfunction. While LVEF remains a critical standard for diagnosing overt CTRCD, its lack of independent predictive value in our adjusted models reinforces the notion that, for the purpose of *risk stratification*, GLS provides a more informative and dynamic assessment of myocardial vulnerability.

A key finding of our study is the delineation of a consistent temporal sequence in the development of anthracycline-induced cardiotoxicity: hs-cTnI elevation precedes GLS worsening by a median of 8.4 weeks, and GLS worsening precedes LVEF decline by a median of 12.6 weeks. This provides a cumulative “window of opportunity” of approximately 21 weeks from first evidence of subclinical injury to the development of overt systolic dysfunction.

This temporal pattern has important clinical implications. First, it supports the use of hs-cTnI as an early “sentinel” biomarker that can trigger intensified surveillance or preventive interventions before functional impairment develops (41). Second, it validates GLS as an intermediate marker that captures the transition from myocardial injury to mechanical dysfunction, aligning with current guideline recommendations for GLS monitoring (14, 42). Third, it suggests that interventions initiated during this window—such as angiotensin-converting enzyme inhibitors, beta-blockers, or dexrazoxane—may have the greatest potential to prevent or reverse cardiotoxicity (43).

Landmark analyses further refined this concept by demonstrating that the combination of early hs-cTnI elevation and GLS worsening identifies an ultra-high-risk subgroup with >58% subsequent CTRCD risk. This finding supports the potential value of a dynamic, risk-adapted surveillance approach, in which patients with early abnormalities may warrant closer monitoring and consideration of cardioprotective therapy. Whether patients without such abnormalities can safely undergo less intensive follow-up requires prospective evaluation.

We recognize that frontline anthracycline use in multiple myeloma differs across regions, being more frequent in China. However, the MY-CARD score's biological rationale is universally relevant and generalizable to any anthracycline-treated malignancy, including breast cancer, sarcoma, and lymphoma. The MY-CARD score was designed to balance statistical rigor with clinical practicality. By selecting three readily available parameters (hs-cTnI, GLS, CAC score) and dichotomizing them at clinically meaningful thresholds—hs-cTnI ≥0.02 μg/L (derived from ROC analysis), GLS > −18% (the guideline-recommended normal value) (42), and CAC score ≥100 Agatston units (established cardiovascular risk threshold) (44)—we created a simple integer-based score ranging from 0 to 6.

The score demonstrated excellent discrimination (C-index 0.88) and effectively stratified risk across all prespecified subgroups. The graded relationship between MY-CARD score and CTRCD incidence—from 6.2% in low-risk to 71.4% in high-risk patients—provides clinically meaningful separation that can guide decision-making. The graded separation in risk suggests that the MY-CARD score may help inform the intensity of surveillance and identify patients who could benefit from early cardio-oncology consultation. However, the optimal monitoring intervals and the role of prophylactic cardioprotective therapy should be established in prospective interventional studies.

The association of the MY-CARD score with MACE and all-cause mortality suggests that its prognostic relevance may extend beyond imaging-defined CTRCD. Each 1-point increase in MY-CARD score was associated with an 86% higher risk of MACE and 72% higher risk of mortality, supporting its use for long-term prognostic stratification in MM survivors.

Several risk stratification tools have been developed for anthracycline-induced cardiotoxicity, primarily in breast cancer populations. The CREC (Cardiotoxicity Risk Evaluation in breast Cancer) model incorporates clinical risk factors and baseline LVEF but does not include GLS or biomarkers. The HFA-ICOS risk score combines demographic, clinical, and cardiac parameters but has limited validation in MM (14). More recently, metabolomic risk scores have shown promise in identifying patients at risk for proteasome inhibitor-related cardiotoxicity (5), but their application to anthracycline toxicity requires further study. Direct comparison with the HFA-ICOS score was not possible in this retrospective study due to incomplete data on all its components. However, our data indicate that objective imaging and biomarker parameters add substantial incremental value over clinical risk factors alone, and future prospective studies are warranted to directly compare the MY-CARD score with the standard HFA-ICOS tool. Our findings provide the essential effect-size estimates and demonstrate the feasibility of integrating these three objective markers into routine practice, thereby establishing a robust methodological and clinical foundation for designing an adequately powered head-to-head comparative effectiveness trial against the HFA-ICOS proforma.

Our study advances the field in several ways. First, it specifically addresses MM patients, who differ from breast cancer populations in age distribution, comorbidity burden, and concurrent therapies (including proteasome inhibitors that may have synergistic cardiotoxic effects) (45). Second, it integrates three complementary domains—myocardial injury (hs-cTnI), mechanical function (GLS), and vascular substrate (CAC)—providing a more comprehensive assessment than any single marker. Third, it establishes a temporal framework that enables dynamic risk reassessment during treatment, rather than relying solely on baseline prediction.

The independent predictive value of CAC score in our study merits mechanistic consideration. Coronary artery calcium reflects cumulative exposure to traditional cardiovascular risk factors and is associated with systemic inflammation, endothelial dysfunction, and impaired coronary flow reserve (46). In the context of anthracycline therapy, pre-existing atherosclerotic burden may amplify cardiotoxicity through several mechanisms: (1) reduced myocardial perfusion reserve may limit compensatory responses to anthracycline-induced metabolic stress; (2) endothelial dysfunction may exacerbate microvascular injury from reactive oxygen species; and (3) shared pathways—such as inflammation and oxidative stress—may be upregulated in both atherosclerotic and anthracycline-injured myocardium (39, 47). This pre-existing vascular vulnerability may be particularly relevant in multiple myeloma patients, who frequently receive concurrent proteasome inhibitors such as bortezomib or carfilzomib (48). These agents are associated with their own cardiovascular toxicities, including hypertension, heart failure, and vasospasm, likely through mechanisms involving endothelial dysfunction and impaired nitric oxide bioavailability (45). We hypothesize that an elevated baseline CAC score, reflecting advanced systemic atherosclerosis, may lower the threshold for clinical decompensation when a patient is exposed to the combined cardiovascular stress of anthracyclines and proteasome inhibitors. This synergistic interaction underscores the importance of comprehensive baseline vascular assessment in this high-risk population.

The finding that hs-cTnI elevation precedes functional decline is consistent with non-ischemic myocardial injury resulting from direct anthracycline-induced cardiomyocyte toxicity, characterized by oxidative stress, topoisomerase II*β* inhibition, and mitochondrial dysfunction, rather than acute coronary thrombosis (30). This mechanistic distinction from type I (ischemic) injury may explain why CAC score provides additive information—it captures the underlying atherosclerotic substrate upon which this direct, non-ischemic myocardial injury is superimposed.

Our findings suggest several potential clinical applications. First, integrating hs-cTnI, GLS, and CAC score at baseline may provide a more comprehensive cardiac risk profile than conventional assessment alone, enabling earlier identification of patients with heightened vulnerability to anthracycline-induced injury. Second, the established temporal sequence supports dynamic risk reassessment during treatment, with early changes in hs-cTnI or GLS potentially guiding surveillance intensity. Third, the MY-CARD score offers a simple, integer-based tool that can facilitate communication of relative risk among oncologists, cardiologists, and patients, thereby supporting more informed, shared decision-making regarding the intensity of cardiac surveillance and the need for cardio-oncology referral.

This study has several limitations. First, its retrospective single-center design may have introduced selection bias, information bias, and residual confounding. Second, although temporal validation was conducted in a later cohort from the same institution, this should not be interpreted as strict independent external validation; therefore, the broader generalizability of the MY-CARD score remains to be established. Third, the validation cohort was relatively small, which may have limited the robustness of validation estimates. Fourth, the overall sample size was moderate, and some subgroup and landmark analyses may have been underpowered or vulnerable to model overfitting. Fifth, CAC scoring is not yet routinely available across all centers, which could constrain the immediate applicability of this strategy in resource-limited settings. Finally, because this was an observational study, we cannot determine whether MY-CARD-guided surveillance or cardioprotective intervention improves clinical outcomes. Accordingly, larger multicenter studies with independent external validation are warranted to confirm the clinical utility of this multi-domain approach and to establish optimal risk-based monitoring protocols.

## 5 Conclusions

In this retrospective study of patients with multiple myeloma receiving anthracycline-based therapy, the combination of hs-cTnI, GLS, and CAC score improved risk stratification for CTRCD compared with conventional assessment alone. The MY-CARD score may provide a practical framework for baseline and early on-treatment risk assessment, with the potential to identify patients who warrant closer cardiac surveillance. Early biomarker elevation and GLS worsening appeared to precede overt dysfunction, suggesting a potentially actionable interval for reassessment during treatment. These findings support further external validation of this multi-domain strategy and prospective studies to determine whether MY-CARD-guided monitoring can improve clinical outcomes.

## Data Availability

The raw data supporting the conclusions of this article will be made available by the authors, without undue reservation.
